# The Decimal System of Notation

**Published:** 1878-08

**Authors:** T. F. Brownell


### The Decimal System of Notation.

Ten is, theoretically ill suited for the radix of a system of notation, because it permits of only one bisection. The half of it is five, an odd number. It also is incapable of any other division. On account of these defects the system is ill adapted to the operations of the shop and market. Although our calculations are universally made in the decimal system, none of our tables of weights and measures are decimal in any one of their subdivisions. In all departments of trade the current prices have been derived from a process of successive halvings. The shopman reckons by halves, quarters, eighths, sixteenths, and thirty-seconds, and not by fifths or tenths. The yardstick is divided in its practical use into halves, quarters, eighths, etc., by successive bisections. Even the sixteenth of a unit is more commonly used in trade than the tenth. In the stock-exchange, shares change in price by eighths of a dollar, and not by tenths. Even with our decimal system of money, we require coins for half and quarter of a dollar for practical use in trading. Almost the entire price-list of our stores advances and recedes by these fractions of a unit formed by successive bisections.

The attempt by the French to compel the use of the decimal system shows the difficulty of such an undertaking. Popular necessities compelled the introduction of binal divisions. The prices of their money and stock markets are still frequently quoted in quarters and eighths. The attempt to divide time decimally was a failure. After trying to give to their decimal metrology a universal application, they have been compelled to modify it in many of their weights and measures. From the inherent defects of a ten scale, all attempts to introduce an international decimal system of weights and measures have met with strong opposition.—T. F. BROWNELL, *in Popular Science Monthly for August.*

